# Supplementary material for: Empirical methods for controlling false positives and estimating confidence in ChIP-Seq peaks
Source: BMC Bioinformatics. 2008 Dec 5;9:523. doi: 10.1186/1471-2105-9-523 (PMC2628906; doi:10.1186/1471-2105-9-523)
Supplement: Additional File 1 — A variety of html documents from the USeq web site detailing the available applications, their best usage, output file type descriptions, command line menus, etc. [file 1471-2105-9-523-S1.zip › USeqUserGuides/JavaDoc.zip!/JavaDoc/index-files/index-20.html]

U-Index


|  |  |  |
| --- | --- | --- |
| **Overview** \| Package \| Class \| Use \| **Tree** \| **Deprecated** \| **Index** \| **Help** | |  |
| **PREV LETTER**   **NEXT LETTER** | **FRAMES**    **NO FRAMES**     **All Classes** |  |


A B C D E F G H I J L M N O P Q R S T U V W X Z 

---


## **U**

**UCSCGeneLine** - Class in util.bio.parsers: **UCSCGeneLine(String, Coordinate)** - Constructor for class util.bio.parsers.UCSCGeneLine: **UCSCGeneLine(String[], int, boolean)** - Constructor for class util.bio.parsers.UCSCGeneLine: **UCSCGeneLineComparator** - Class in util.bio.parsers: **UCSCGeneLineComparator()** - Constructor for class util.bio.parsers.UCSCGeneLineComparator: **UCSCGeneLineComparatorScore** - Class in util.bio.parsers: Sorts by score **UCSCGeneLineComparatorScore(int)** - Constructor for class util.bio.parsers.UCSCGeneLineComparatorScore: **UCSCGeneModelTableReader** - Class in util.bio.parsers: Parses a UCSC gene model table file (either refSeq or refFlat), use table options for #hg17.refGene.name hg17.refLink.product hg17.geneName.name hg17.refSeqSummary.summary... **UCSCGeneModelTableReader(File, int)** - Constructor for class util.bio.parsers.UCSCGeneModelTableReader: **UCSCGeneModelTableReader()** - Constructor for class util.bio.parsers.UCSCGeneModelTableReader: **UNIT\_TAG** - Static variable in class edu.utah.seq.parsers.BarParser: **updateAntibody(AntibodyBean, UserBean, DBUtil)** - Static method in class bioroot.antibody.AntibodyModify: Attempts to update a AntibodyBean in the database, only checks that the name is unique if it has changed. **updateAntibodyPreps(AntibodyBean, DBUtil)** - Static method in class bioroot.antibody.AntibodyModify: Attempts to update the AntibodyPreps and UseDilutions in the database. **updateCommonAccessFields(AntibodyPrep[], DBUtil)** - Static method in class bioroot.antibody.AntibodyModify: Just update the commonly accessible fields in each antibodyPrep as well as the useDilutions. **updateCommonAccessFields(DBUtil)** - Method in class bioroot.antibody.AntibodyPrep: Just updates fields that are accessible by all lab group memebers. **updateCommonAccessFields(ReagentBean, String)** - Method in class bioroot.DBUtil: Just updates fields that are accessible by all lab group memebers. **updateCommonAccessFields(ReagentBean, UserBean, HttpServletRequest)** - Method in class bioroot.DBUtil: Just update the commonly accessible fields: lastUser, comments **updateCommonAccessFields(DBUtil, String)** - Method in class bioroot.ReagentBean: Just updates fields that are accessible by all lab group memebers, lastUser, comments given a reagent type: Oligo or Plasmid. **updateConsole()** - Method in class gata.plotter.Console: **updateConsole()** - Method in class util.apps.gump.GumpResults: **updateConsole()** - Method in class util.apps.gump.GumpResultsScrollPane: **updateLoginFailure(String)** - Method in class bioroot.DBUtil: Increments the numberFailures for an ip in the Login table if it exists or creates a new record. **updateLoginFailure(String)** - Method in class cgar.Util: Increments the numberFailures for an ip in the Login table if it exists or creates a new record. **updateOld(DBUtil, UserBean)** - Method in class bioroot.antibody.AntibodyBean: Updates a Antibody entry, don't forget to update geneId **updateOld(DBUtil)** - Method in class bioroot.antibody.AntibodyPrep: Updates a Antibody prep entry **updateOld(DBUtil)** - Method in class bioroot.antibody.UseDilution: **updateOld(DBUtil, UserBean)** - Method in class bioroot.CellTypeBean: **updateOld(DBUtil, UserBean)** - Method in class bioroot.GeneBean: **updateOld(DBUtil, UserBean)** - Method in class bioroot.MarkerBean: **updateOld(DBUtil, UserBean)** - Method in class bioroot.oligo.OligoBean: **updateOld(DBUtil, UserBean)** - Method in class bioroot.OrganismBean: **updateOld(DBUtil, UserBean)** - Method in class bioroot.plasmid.PlasmidBean: **updateOld(DBUtil, UserBean)** - Method in class bioroot.strain.StrainBean: Updates a Strain entry, don't forget to update markers and plasmids. **updateOligo(OligoBean, UserBean, HttpServletRequest, DBUtil)** - Static method in class bioroot.oligo.OligoModify: Attempts to update an OligoBean in the database, only checks that the name is unique if it has changed. **updatePlasmid(PlasmidBean, UserBean, HashMap, HttpServletRequest, DBUtil)** - Static method in class bioroot.plasmid.PlasmidModify: Attempts to update a PlasmidBean in the database, only checks that the name is unique if it has changed. **updateStrain(StrainBean, UserBean, HashMap, HttpServletRequest, DBUtil)** - Static method in class bioroot.strain.StrainModify: Attempts to update a StrainBean in the database, only checks that the name is unique if it has changed. **updateStrainCommonAccessFields(DBUtil)** - Method in class bioroot.strain.StrainBean: Just updates fields that are accessible by all lab group memebers. **updateStrainCommonAccessFields(StrainBean, UserBean, HashMap, DBUtil)** - Static method in class bioroot.strain.StrainModify: Just update the commonly accessible fields: restock, lastUser, remainingAliquots, comments **updateSucessfulLogin(SQL, String)** - Method in class cgar.beans.LoginBean: Updates the User and Login tables to reflect a successful login. **updateUser()** - Method in class bioroot.UserRegistrationBean: **updateUserLabGroupTable(int, int, String)** - Method in class bioroot.UserRegistrationBean: **upload(File[])** - Method in class util.amazon.S3Manager: Upload files to S3. **upload(String, File[])** - Method in class util.amazon.S3Manager: Uploads files to S3 using a different thread. **upload(String, File)** - Method in class util.amazon.S3Manager: See threaded upload (String uniqueProcessName, File[] files). **upload(File)** - Method in class util.amazon.S3Manager: See upload (File[] files). **UseDilution** - Class in bioroot.antibody: **UseDilution(ResultSet, int)** - Constructor for class bioroot.antibody.UseDilution: **UseDilution()** - Constructor for class bioroot.antibody.UseDilution: **useDilutionFeatureNames** - Static variable in class bioroot.antibody.AntibodyBase: Used to see if any use dilutions are called for spreadsheet. **UserBean** - Class in bioroot: User information. **UserBean()** - Constructor for class bioroot.UserBean: **UserBean(DBUtil, LoginBean)** - Constructor for class bioroot.UserBean: **UserBean** - Class in cgar.beans: User info, where possible this should come from GNomEx, use U ids/ passwords, when not available store md5 salted encrypted passwords, do not use db specific encryption function, use Nix's MD5Crypt.java class. **UserBean()** - Constructor for class cgar.beans.UserBean: **UserBean(Util, LoginBean)** - Constructor for class cgar.beans.UserBean: **UserRegistration** - Class in bioroot: **UserRegistration()** - Constructor for class bioroot.UserRegistration: **UserRegistrationBean** - Class in bioroot: **UserRegistrationBean()** - Constructor for class bioroot.UserRegistrationBean: **UserRegistrationBean(UserBean)** - Constructor for class bioroot.UserRegistrationBean: **UserTypeBean** - Class in cgar.beans: User type for defining access/ permissions: CGAR Administrator - no restrictions Group Super User - no restrictions on view and edit within the lab Group Member - view there own and other group items, only edit there own Group Guest - only group view privileges, no edit Anonomous - no edit, only view www A user type is specific for a particular lab. **UserTypeBean()** - Constructor for class cgar.beans.UserTypeBean: **Util** - Class in bioroot: Utility class for BioRoot **Util()** - Constructor for class bioroot.Util: **Util** - Class in cgar: **Util()** - Constructor for class cgar.Util: **Util** - Class in trans.misc: Static helper methods for all things Affy **Util()** - Constructor for class trans.misc.Util: **util.amazon** - package util.amazon: **util.apps** - package util.apps: Scripts for printing arrays, manipulating files, scatter plots... **util.apps.gump** - package util.apps.gump: **util.bio.annotation** - package util.bio.annotation: For representing gene annotation. **util.bio.calc** - package util.bio.calc: Calculators for aligments and nucleic acids. **util.bio.cluster** - package util.bio.cluster: Clustering apps and methods. **util.bio.converters** - package util.bio.converters: Bio format converters. **util.bio.digest** - package util.bio.digest: Restriction enzyme digestion of DNA sequences. **util.bio.parsers** - package util.bio.parsers: Parsers and extractors, fasta, quality, text, gff.... **util.bio.parsers.gff** - package util.bio.parsers.gff: GFF2 and GFF3 parsers. **util.bio.seq** - package util.bio.seq: Nucleic acid sequence manipulation. **util.bio.wrappers** - package util.bio.wrappers: Wrappers for other bio applications. **util.bio.xamer** - package util.bio.xamer: Generation and manipulation of random sequence words of various lengths. **util.gen** - package util.gen: Variety of helper methods for Email, IO, Encryption, HTML, Swing... **util.xml** - package util.xml: **UtilMeme** - Class in meme: A catch all class to hold static methods and parameters set by different classes associated by MemeR. **UtilMeme()** - Constructor for class meme.UtilMeme: **UtilSelex** - Class in selex: Utilites for the Selex package. **UtilSelex()** - Constructor for class selex.UtilSelex

---


|  |  |  |
| --- | --- | --- |
| **Overview** \| Package \| Class \| Use \| **Tree** \| **Deprecated** \| **Index** \| **Help** | |  |
| **PREV LETTER**   **NEXT LETTER** | **FRAMES**    **NO FRAMES**     **All Classes** |  |


A B C D E F G H I J L M N O P Q R S T U V W X Z 

---
